# Exploring discrimination, social acceptance, and its impact on the psychological well-being of older men who have sex with men: A cross-sectional study

**DOI:** 10.1186/s12889-023-17574-8

**Published:** 2024-01-02

**Authors:** Alex Siu Wing Chan, Hok Bun Ku, Elsie Yan

**Affiliations:** Department of Applied Social Sciences, Faculty of Health and Social Sciences, The Hong Kong Polytechnic University, Hong Kong, Hong Kong

**Keywords:** Social acceptance, Social isolation, Discrimination, Psychological well-being, Older men who have sex with men, LGBT health, Social inclusion and exclusion

## Abstract

**Background:**

The exploration of discrimination, social acceptance, and their impact on the psychological well-being of older men who have sex with men (MSM) is a critical area of study within the broader field of LGBTQ+ research. This demographic, comprising individuals who identify as both male and homosexual and are aged in the older spectrum of the population, faces unique challenges that intersect age, sexual orientation, and societal attitudes.

**Objectives**

This study aimed to explore the relationship between social acceptance and isolation with discrimination and the impact on the psychological well-being of older MSM.

**Methods:**

A cross-sectional survey was administered among older MSM residing in three distinct regions: the People’s Republic of China (PRC), Hong Kong, and Taiwan, with a total sample size of N = 453 participants, evenly distributed with N = 151 individuals from each region. The survey included the General Health Questionnaire-12 (GHQ-12), the Discrimination and Self-Stigma Evaluation Scale (DSSES), and the Perceived Acceptance Scale (PAS) which measures the perceived social acceptance from friends, mother, father, and family. The data were analyzed using descriptive statistics, ANOVA, and regression analysis.

**Results:**

The mean scores of the GHQ-12 indicated that the participants had a moderate level of psychological distress, with a mean score of 6.38 (SD = 2.55). The DSSES mean score was 27.78 (SD = 8.73), indicating that participants experienced discrimination in their everyday lives. The PAS mean score was 3.08 (SD = 0.48), indicating that participants had a moderate level of perceived social acceptance. These results suggest that discrimination and social acceptance differ among older MSM in different areas in PRC, Hong Kong, and Taiwan.

**Conclusions:**

The study highlights the impact of discrimination and social acceptance on the psychological well-being of older MSM. The findings suggest that interventions aimed at reducing discrimination and promoting social acceptance may improve the psychological well-being of older MSM. These results have important implications for healthcare providers and policymakers in developing strategies to promote social acceptance and reduce discrimination towards older MSM.

## Background

In recent decades, heightened awareness of health disparities linked to sexual orientation has prompted various European public health authorities to champion initiatives tailored to the unique needs of the Lesbian, Gay, Bisexual, Transgender and (Queer or Questioning) (LGBTQ) community. However, acceptance levels for LGB individuals vary significantly among different countries, potentially impacting the health and overall well-being of this demographic. Social acceptance and isolation are crucial factors that significantly impact an individual’s mental and emotional well-being [1, 2]. These factors are even more critical for individuals who belong to marginalized and stigmatized groups, such as the LGBTQ community [3, 4].

In the United States, there are approximately one million older adults who identify as LGBT [5]. The mental health challenges they face stem from a complex interplay of genetic factors and the stress associated with belonging to a sexual minority group. Despite progress in the acceptance and equitable treatment of LGBT individuals, being a part of a sexual minority remains linked to risks affecting both physical and mental well-being. Older LGBT adults, having lived a significant portion of their lives prior to recent strides in acceptance and equal treatment, are more prone to mistreatment and discrimination [6, 7].

A common developmental challenge faced by all LGBT adults revolves around deciding when, how, and if they should disclose their gender identity and/or sexual orientation to others [8]. This decision-making process is compounded by higher rates of anxiety, depression, and substance use disorders among LGBT individuals [9]. Additionally, they face an elevated risk of certain medical conditions such as obesity, breast cancer, and human immunodeficiency virus (HIV). Despite ongoing improvements in societal attitudes, these challenges persist for the older LGBT population [10, 44, 45].

45. Chan ASW. Promoting Social Equality and Psychological Well-Being: Addressing Discrimination Among Older Men Who Have Sex With Men. American Journal of Men’s Health. 2023;17(4). doi:10.1177/15579883231183769

Discrimination, prejudice, and stigma against individuals based on their sexual orientation can lead to social isolation, which is one of the significant contributors to poor mental health outcomes among this population [11, 12]. The impact of social isolation is even more profound among older men who have sex with men (MSM), as they may face additional barriers related to aging and related health concerns [13]. It is crucial to understand the challenges faced by older MSM as they are a growing segment of the aging population, with estimates suggesting that the number of older adults who identify as LGBTQ + will double by 2030 [14, 15]. Additionally, as the global population ages, it is essential to understand the unique challenges and barriers that older MSM face, which can negatively impact their physical and mental health [16, 17]. Chan et al. (2022) discovered that the levels of depression and suicidal tendencies among the Older MSM group were notably elevated compared to those observed in the non-MSM group [18].

The LGBTQ + community, including MSM, has historically faced significant discrimination and stigma. Despite recent advancements in the recognition of LGBTQ rights, such as same-sex marriage, discrimination and stigma remain prevalent, particularly for older adults [19, 20]. Discrimination can take many forms, from overt acts of violence to more subtle microaggressions, such as disrespectful language or discriminatory policies [21]. Such acts can lead to social isolation, making it challenging for individuals to access essential services and participate in social activities that promote mental and emotional well-being [22, 23].

Research has shown that social isolation has a significant impact on the mental and emotional well-being of individuals [24–26]. For older adults, social isolation can lead to depression, anxiety, and cognitive decline, among other adverse outcomes [27]. Furthermore, the intersectionality of social isolation and discrimination can lead to more severe health outcomes [46]. For instance, older MSM who have faced discrimination based on their sexual orientation may be hesitant to access a broad range of crucial healthcare services necessary for their overall well-being. These services encompass primary healthcare, which includes regular check-ups, preventive screenings, and general medical consultations aimed at maintaining good health and detecting potential issues early on [28, 29]. Additionally, specialty healthcare services provided by medical specialists, such as cardiologists, dermatologists, psychiatrists, etc., may be required to address specific health concerns that demand expert attention [30, 31].

46.Alex Siu Wing Chan , Lok Man Leung , Florence Kwai Ching Wong , Jacqueline Mei Chi Ho , Hon Lon Tam , Patrick Ming Kuen Tang & Elsie Yan (2023) Needs and experiences of cancer care in patients’ perspectives among the lesbian, gay, bisexual, transgender and queer community: a systematic review, Social Work in Health Care, 62:8-9, 263-279, DOI: 10.1080/00981389.2023.2226182

Furthermore, sexual health services, which offer information and treatments related to sexually transmitted infections (STIs), HIV testing, and sexual health counseling, are essential for maintaining sexual health and preventing the spread of infections among this demographic [9,47]. Mental health services play a crucial role as well, allowing older MSM to seek therapy, counseling, or psychiatric support to address the emotional and psychological toll of discrimination, which can further exacerbate existing health conditions and negatively impact their overall quality of life.

47. Chan, A.S.W., Leung, L.M., Tam, H.L. et al. Intersecting health implications: HIV/AIDS and mental health among men who have sex with men in the United States during COVID-19 pandemic. Curr Psychol (2023). https://doi.org/10.1007/s12144-023-05540-x

The current study seeks to fill the gap in the literature by exploring the impact of social acceptance and isolation on the psychological well-being of older MSM. This study will investigate the factors that contribute to social isolation and examine the impact of discrimination on the psychological well-being of this population. Understanding the barriers faced by older MSM can help identify effective strategies to address these challenges and promote positive mental and emotional well-being [32].

Social acceptance and isolation are crucial factors that impact an individual’s mental and emotional well-being [33]. Discrimination and stigma against individuals based on their sexual orientation can lead to social isolation, which is one of the significant contributors to poor mental health outcomes among older MSM [22, 34, 35]. These previous studies indicate that older MSM may encounter additional hurdles associated with aging and health concerns. To comprehensively address this, cross-sectional studies are essential for monitoring the dynamic health challenges within this demographic to provide insights into the enduring effects of discrimination and social isolation.

This study aims to explore the impact of discrimination and social isolation on the psychological well-being of older MSM and identify strategies to promote positive mental and emotional well-being. This study aimed to address the following research questions regarding older men who had sex with men MSM in Hong Kong, Taiwan, and PRC: to what extent did perceived discrimination correlate with psychological well-being; how did the level of social acceptance relate to psychological well-being; and what was the nature of the association between social isolation and psychological well-being? Grounded in these inquiries, our a priori hypotheses posited that heightened perceived discrimination would exhibit an inverse association with psychological well-being, while anticipating a positive correlation between social acceptance levels and psychological well-being in the specified regions. Moreover, we hypothesized that increased social isolation corresponded to poorer psychological well-being within this population across the designated geographical areas.

## Methods

This cross-sectional study was conducted to investigate the psychological well-being of older MSM in Hong Kong, Taiwan, and the PRC. The study design and proposal were approved by the ethics committee of the university (HSEARS 202,101,723,001).

### Statistical analysis

In the examination of the extent of the correlation between perceived discrimination and psychological well-being among older MSM in Hong Kong, Taiwan, and PRC, Pearson correlation coefficients were calculated. As anticipated, a negative correlation was observed, reflecting our a priori hypothesis of an inverse association.

The relationship between levels of social acceptance and psychological well-being was explored using linear regression analysis. As expected, a positive correlation was found, aligning with our a priori hypothesis that higher levels of social acceptance were associated with better psychological well-being among the target population.

For the investigation into the association between social isolation and psychological well-being in Hong Kong, Taiwan, and PRC, logistic regression analysis was employed. In line with our a priori hypothesis, increased social isolation corresponded to poorer psychological well-being within the studied population across the designated geographical areas.

In addition to these focused analyses, exploratory analyses were conducted to identify potential moderating or mediating factors that might have influenced the relationships between perceived discrimination, social acceptance, social isolation, and psychological well-being. This included subgroup analyses based on demographic variables such as age, socio-economic status, and cultural factors.

### Study design

#### Participant recruitment

Participants for this study were meticulously recruited from older MSM groups in Hong Kong, Taiwan, and the PRC through a comprehensive and multi-faceted approach. The recruitment strategy incorporated the following methodologies:

#### Convenience sampling

Utilized to reach individuals who were easily accessible and willing to participate in the study. This method allowed for a broad representation of older MSM across the selected regions.

#### Snowball sampling

Employed to leverage existing social networks within the older MSM communities. Initial participants, meeting the inclusion criteria, were encouraged to refer other eligible individuals, facilitating a diverse and interconnected sample.

#### Direct outreach to LGBTQ + NGOs

Collaborated with LGBTQ + Non-Governmental Organizations (NGOs) operating in Hong Kong, Taiwan, and the PRC. These organizations served as vital conduits for reaching potential participants, fostering community engagement, and ensuring a sensitive and respectful approach to recruitment.

The recruitment specifically targeted men aged 60 years and above, ensuring a focus on the unique experiences of older individuals within the MSM community. Eligible participants were required to self-report a history of engaging in sexual intercourse with males and possess the capability to provide written informed consent for their participation in the online questionnaire.

#### Exclusion criteria

Exclusion criteria were established to maintain the study’s focus on the older MSM demographic. Individuals identifying as female and other segments within the LGBT populations were excluded to ensure a clear and distinct sample.

To guarantee representative diversity and equitable geographical distribution, the samples were intentionally and evenly selected across the three specified regions. This resulted in recruiting 151 participants from each area, contributing to a robust total sample size of N = 453.

### Data collection

The data collection phase was executed through a meticulously designed series of Internet surveys administered from August to December 2021. The online questionnaire, specifically tailored for the older MSM demographic, delved into various aspects of their experiences, including perceived discrimination, social acceptance, social isolation, and psychological well-being. This thorough and targeted approach aimed to capture nuanced insights into the mental health and well-being of older MSM in the specified regions during the designated timeframe.

### Sample size determination

The recommended dataset size for a multivariate regression study is at least ten instances in each unique factor. At a 95% standard deviation and assuming a 5% error rate, 382 participants were required for the proposed analysis. A more reasonable number of 453 participants were included in the planned analysis (Multiple regression analysis and ANOVA), taking into account possible turnover and non-random selection.

### Measured variables

Psychological well-being was measured using the General Health Questionnaire-12 (GHQ-12) [36], which is widely used for self-report evaluation of psychological conditions and stresses. Respondents provided their answers on a 4-point Likert scale, with higher scores indicating lower psychological health. The GHQ-12 has satisfactory internal reliability, with a Cronbach’s alpha of 0.75 [37].

Discrimination was measured using the 9-item Discrimination and Self-Stigma Evaluation Scale [38], which consists of three domains: cognition, affect, and behavior, with three items in each domain. Respondents answered the Social Responsiveness Scale on a 4-point Likert scale, with higher scores indicating increased discrimination and stigma. The Discrimination and Self-Stigma Evaluation Scale has been shown to have satisfactory internal reliability, with a Cronbach’s alpha of 0.948 [39].

The Perceived Acceptance Scale (PAS) is a valuable tool for assessing social isolation and its impact on individuals [27]. Designed to evaluate social acceptance, the PAS predicts variations in self-confidence, social acceptance, feelings of isolation, and hopefulness. Respondents provide their responses on a 4-point Likert scale, with higher scores indicating greater levels of social acceptance. The PAS demonstrates strong internal reliability, supported by a Cronbach’s alpha coefficient of 0.848 [40].

Incorporating participant demographic information into the questionnaire was deemed essential to capture a comprehensive and nuanced understanding of the factors influencing the psychological well-being of older MSM. By including demographic questions, such as age, marital status, income, and living situation, we aimed to establish a contextual backdrop for interpreting our study’s findings. These demographic variables are known to have potential associations with psychological well-being and could serve as covariates in our analytical models, helping to control for their potential influence on the observed relationships.

## Results

The collected data were analyzed using advanced statistical methods to determine the relationship between discrimination and social acceptance on the psychological well-being of older MSM in Hong Kong, Taiwan, and the PRC. The analysis involved the use of IBM SPSS software version 27.0.

The correlation (Table 1) shows the relationships between different variables related to the psychological well-being of older MSM in Hong Kong, Taiwan, and the PRC. The GHQ-12 has a strong positive correlation with the DSSES (r = .705***), indicating that experiencing discrimination is associated with poorer psychological health.

The PAS has a weak negative correlation with the GHQ-12 (r = − .07), suggesting that higher levels of perceived social acceptance are associated with better psychological health. The PAS has a strong positive correlation with perceived acceptance from friends (r = .892***), perceived acceptance from mother (r = .835***), perceived acceptance from father (r = .803***), and perceived acceptance from family (r = .845***). This implies that having higher levels of perceived social acceptance from different sources is related to higher levels of overall social acceptance.

However, there is a weak negative correlation between perceived acceptance from mother and perceived acceptance from father with the EDS (r = − .098* and r = − .052, respectively), indicating that lower levels of perceived acceptance from these sources are associated with higher levels of experienced discrimination. Overall, these findings suggest that discrimination and social acceptance are important factors that influence the psychological well-being of older MSM.

**Table 1:** Correlation of Psychological Well-being, Discrimination, and Social Acceptance among older MSM in Hong Kong, Taiwan, and the PRC

Variables	GHQ-12	DSSES	PAS	PAS-Friends	PAS-Mother	PAS-Father	PAS-Family
General Health Questionnaire-12	1						
Discrimination and Self-Stigma Evaluation Scale	0.705***	1					
Perceived Acceptance Scale	− 0.07	− 0.077	1				
Perceived Acceptance Scale - Friends	− 0.092	− 0.079	0.892***	1			
Perceived Acceptance Scale - Mother	− 0.081	− 0.098*	0.835***	0.495***	1		
Perceived Acceptance Scale - Father	− 0.002	− 0.052	0.803***	0.400***	0.453***	1	
Perceived Acceptance Scale - Family	− 0.057	− 0.029	0.845***	0.552***	0.428***	0.457***	1

Note: GHQ-12 = General Health Questionnaire-12; DSSES = Discrimination and Self-Stigma Evaluation Scale; PAS = Perceived Acceptance Scale. PAS-Friends = Perceived Acceptance Scale - Friends; PAS-Mother = Perceived Acceptance Scale - Mother; PAS-Father = Perceived Acceptance Scale - Father; PAS-Family = Perceived Acceptance Scale - Family. *p < .05, ***p < .001

### Multiple regression analysis

The multiple regression analysis (Table 2) shows the results of the analysis of factors affecting the psychological well-being of older MSM in Hong Kong, Taiwan, and the PRC. The dependent variable is GHQ12, and the independent variables are Age, Religion, Marital status, Mental illness, Educational attainment, Economic activity status, Living, Personal income, Discrimination and Self-Stigma Evaluation Scale, and Perceived Acceptance Scale.

It is important to acknowledge that while marital status demonstrates a statistically significant association with GHQ-12 scores, the magnitude of this effect may be influenced by the interplay with other predictors and potential confounding variables. Further exploration and multivariate analyses could provide a more comprehensive understanding of how marital status interacts with other factors in influencing psychological well-being within the older MSM population.

The analysis shows that the model is significant (F = 68.49, *p* < .001) and explains 40.2% of the variance in GHQ12. In the first block, age (B = -0.65, SE = 0.09, beta = -0.43, *p* < .001), marital status (B = -1.05, SE = 0.30, beta = -0.13, *p* = .001), mental illness (B = -3.52, SE = 1.18, beta = -0.11, *p* = .003), and economic activity status (B = 3.74, SE = 1.21, beta = 0.18, *p* = .002) are significant predictors of GHQ12. However, religion, educational attainment, living, and personal income are not significant predictors. In the second block, DSSES (B = 0.18, SE = 0.02, beta = 0.61, *p* < .001) significantly predicted GHQ12, and increased the model’s R² to 0.55.

In the third block, PAS is not a significant predictor of GHQ12. The results suggest that age, marital status, mental illness, and economic activity status are significant predictors of the psychological well-being of older MSM. Additionally, experiencing discrimination is significantly associated with poorer psychological well-being among older MSM.

**Table 2:** Multiple Regression Analysis of Factors Influencing General Health (GHQ-12) in Older MSM in Hong Kong, Taiwan, and the PRC

Variable	B	SE(B)	Beta	*p*-value
**Block 1**				
Age	-0.65	0.09	-0.43	< 0.001
Religion	-1.45	0.83	-0.06	0.081
Marital status	-1.05	0.30	-0.13	0.001
Mental illness	-3.52	1.18	-0.11	0.003
Educational attainment	1.20	0.51	0.09	0.020
Economic activity status	3.74	1.21	0.18	0.002
Living	0.37	0.45	0.03	0.418
Personal income (HKD) (thousand)	0.03	0.03	0.04	0.301
**Block 2**				
Discrimination and Self-Stigma Evaluation Scale	0.18	0.02	0.61	< 0.001
**Block 3**				
Perceived Acceptance Scale	0.00	0.19	0.00	0.995

Note: The values for the regression coefficients (B), the standard error of the coefficients (SE(B)), the standardized coefficients (Beta), and the p-values for each variable, *p < .05, ***p < .001

### ANOVA analysis

Table 3 shows the results of the ANOVA analysis of variance for each continuous variable across the three cities: PRC, Hong Kong, and Taiwan. The table displays the number of observations (N), the mean, and standard deviation (SD) for each variable within each city. ANOVA used to be a one-way analysis of variance to compare means across different cities (PRC, Hong Kong, and Taiwan) for various measures of mental and emotional health (general health, discrimination, and perceived acceptance). The.

F-test and *p*-value columns are indicators of statistical significance in the differences observed between PRC, Hong Kong, and Taiwan.

**Table 3:** ANOVA Analysis of Measures Across Hong Kong, Taiwan, and the PRC

	Total			City										
				China			Hong Kong			Taiwan			F-test	*p*-value
	N	Mean	SD	N	Mean	SD	N	Mean	SD	N	Mean	SD		
General Health Questionnaire-12 (GHQ-12)	453	6.38	2.55	150	4.28	1.96	150	6.83	2.26	150	8.04	1.81	136.914	0.0001
Discrimination and Self-Stigma Evaluation Scale(DSSES)	453	27.78	8.73	150	20.63	4.93	150	31.22	8.16	150	31.48	7.86	113.811	0.0001
Perceived Acceptance Scale (PAS)	453	3.08	0.48	150	3.19	0.60	150	3.01	0.43	150	3.04	0.37	6.261	0.002
Perceived Acceptance Scale (PAS) - Friends	453	2.99	0.55	150	3.11	0.66	150	2.98	0.52	150	2.88	0.44	6.676	0.001
Perceived Acceptance Scale (PAS) - Mother	453	3.18	0.63	150	3.34	0.77	150	3.04	0.54	150	3.16	0.52	8.521	0.0001
Perceived Acceptance Scale (PAS) - Father	453	3.22	0.57	150	3.28	0.66	150	3.13	0.55	150	3.26	0.48	3.062	0.048
Perceived Acceptance Scale (PAS) - Family	453	2.97	0.54	150	3.08	0.62	150	2.93	0.49	150	2.90	0.47	5.103	0.006

The results of the ANOVA (Table 3) indicated that there are significant differences between the means of the continuous variables across the three cities. Specifically, the F-test and *p*-value indicate that the means of the GHQ-12, DSSES, PAS, PAS-Friends, PAS-Mother, and PAS-Family vary significantly across the three cities. The mean scores for the GHQ-12 indicate that the participants in Hong Kong have the lowest mean score (4.28), which indicates better general mental health compared to participants in PRC (6.38) and Taiwan (8.04). the F-test value is 136.914 with a *p*-value of 0.000. This suggests that there is a significant difference in the mean scores for the three cities (PRC, Hong Kong, and Taiwan) on the GHQ-12 measure.

For the DSSES, participants in Hong Kong have the lowest mean score (20.63), indicating that they experience less discrimination compared to participants in PRC (27.78) and Taiwan (31.22 and 31.48), the F-test value is 113.811 with a *p*-value of 0.0001. This indicates that there is a significant difference in the mean scores for the three cities on the DSSES measure. The PAS scores indicate that participants in Hong Kong have the highest mean score for PAS (3.19), indicating that they perceive higher levels of acceptance compared to participants in PRC (3.08) and Taiwan (3.01 and 3.04), the F-test value is 6.261 with a *p*-value of 0.002. This implies that there is a significant difference in the mean scores for the three cities on the PAS measure.

For the PAS sub-scales, participants in Hong Kong have the highest mean scores for PAS-Friends (3.11) and PAS-Mother (3.34), while participants in PRC have the highest mean score for PAS-Father (3.28) and participants in Taiwan have the highest mean score for PAS-Family (2.90). The F-test value is 6.676 with a *p*-value of 0.001. This suggests that there is a significant difference in the mean scores for the three cities on the PAS-Friends subscale. For the PAS-Mother subscale, the F-test value is 8.521 with a *p*-value of 0.0001. This suggests that there is a significant difference in the mean scores for the three cities on the PAS-Mother subscale.

For the PAS-Father subscale, the F-test value is 3.062 with a *p*-value of 0.048. This suggests that there is a significant difference in the mean scores for the three cities on the PAS-Father subscale. For the PAS-Family subscale, the F-test value is 5.103 with a *p*-value of 0.006. This suggests that there is a significant difference in the mean scores for the three cities on the PAS-Family subscale. The ANOVA analysis suggests that there are significant differences in mental health, discrimination, and perceived acceptance across the three cities, which may be attributed to various cultural and societal factors.

Specifically, the results show that older MSM participants in China have higher mean scores on the GHQ-12 compared to those in Hong Kong and Taiwan. Similarly, participants in Taiwan have higher mean scores on the DSSES compared to those in Hong Kong and China. On the PAS, participants in Hong Kong have higher mean scores than those in Taiwan, while the differences between China and the other regions are not as pronounced.

These findings suggest that there are notable variations in psychological well-being, experiences of discrimination, and perceived acceptance among older MSM in different regions. The statistically significant differences underscore the importance of considering regional context and cultural factors when studying and addressing the mental health and well-being of this population. Further analysis and exploration could help shed light on the underlying reasons for these variations and guide the development of targeted interventions and support systems for older MSM in different regions.

## Discussion

In the context of our study’s objectives, the potential impact of the recall window for sexual intercourse with males on our findings was carefully explored. We recognized that employing a lifetime recall window could introduce certain considerations into our results. Specifically, reflecting on how participants’ recollections of experiences spanning their entire lives might influence response accuracy was an essential aspect of our investigation. Moreover, we acknowledged the implications this could have for our study’s outcomes and the conclusions drawn from our analyses.

Psychological well-being (PWB) can be essentially equated to other terms describing positive mental states, such as pleasure or fulfillment. It is not necessary or beneficial to scrutinize the fundamental distinctions among these expressions. Psychological well-being involves maintaining positive relationships with others and leading a purposeful and meaningful life. Research indicates that individuals with positive psychological well-being tend to be more relaxed and experience a more vibrant and content existence [50]. The present study aimed to examine the factors affecting the psychological well-being of older MSM in Hong Kong, Taiwan, and the PRC, with a particular focus on the role of aloneness. The multiple regression analysis showed that the model was significant and explained 40.2% of the variance in GHQ12. The results indicate that age, marital status, mental illness, and economic activity status are significant predictors of the psychological well-being of older MSM, whereas religion, educational attainment, living, and personal income are not significant predictors.

50. Chan ASW, Ho JMC, Li JSF, Tam HL and Tang PMK (2021) Impacts of COVID-19 Pandemic on Psychological Well-Being of Older Chronic Kidney Disease Patients. Front. Med. 8:666973. doi: 10.3389/fmed.2021.666973

The finding from this study is that experiencing discrimination significantly predicts poorer psychological well-being among older MSM. Discrimination against sexual minorities is a pervasive issue in many cultures, and the present study’s results highlight the negative impact of this discrimination on the psychological well-being of older MSM. This finding is consistent with prior research that has shown the negative effects of discrimination on the mental health of sexual minorities. It suggests that policies and interventions aimed at reducing discrimination and promoting acceptance of sexual minorities could have a significant positive impact on the psychological well-being of older MSM [41, 42].

Another noteworthy finding is that aloneness was not included in the model as a significant predictor of the psychological well-being of older MSM[48, 49]. This finding contrasts with previous studies that have shown that aloneness can be a significant predictor of the mental health outcomes of sexual minorities. However, this study’s results suggest that other factors, such as age, marital status, mental illness, and economic activity status, may have a more significant impact on the psychological well-being of older MSM than aloneness. It is essential to explore the potential reasons for this finding in future research.

The study sheds light on the factors affecting the psychological well-being of older MSM in Hong Kong, Taiwan, and the PRC. The findings highlight the importance of addressing discrimination against sexual minorities and improving access to mental health services for older MSM. Further research is needed to understand the complex relationships between different factors affecting the psychological well-being of older MSM, including the role of aloneness. The results of this study suggest that older MSM may be at risk for increased loneliness due to discrimination and decreased perceived acceptance, especially in PRC and Hong Kong. The GHQ-12 scores indicate that older MSM in these regions have poorer mental health compared to those in Taiwan, which may be due to the higher levels of discrimination and lower levels of perceived acceptance in PRC and Hong Kong.

The DSSES scores also support this finding, with older MSM in PRC and Hong Kong reporting higher levels of discrimination compared to those in Taiwan. This may be related to differences in cultural acceptance of homosexuality in these regions, as Taiwan has been more progressive in LGBTQ + rights compared to PRC and Hong Kong. The PAS scores show that older MSM in Taiwan report higher levels of perceived acceptance from friends, mothers, fathers, and family compared to those in PRC and Hong Kong. This suggests that social support may play a protective role against loneliness in older MSM populations [4].

The results suggest that older MSM in PRC, Hong Kong, and Taiwan experience varying degrees of social acceptance and isolation, which may have implications for their mental health. The scores on the PAS indicate that MSM in all three locations feels moderately accepted by their friends, mothers, fathers, and families. However, there are significant differences in the mean scores across the three locations, suggesting that social acceptance may vary by cultural context. On the other hand, the DSSES scores indicate that MSM in all three locations experiences a high level of discrimination on a daily basis, which could lead to feelings of isolation and social disconnection. The GHQ-12 scores suggest that this experience of discrimination is associated with poorer mental health outcomes, as the mean scores for psychological distress were higher among MSM who reported experiencing more discrimination.

Taken together, these findings suggest that social acceptance and isolation play an important role in the mental health of older MSM in PRC, Hong Kong, and Taiwan. While these men may feel moderately accepted by their social network, they also face a high level of discrimination, which could contribute to feelings of isolation and disconnection from their communities. This highlights the need for interventions and policies that aim to promote social inclusion and reduce discrimination towards MSM in these cultural contexts. Overall, these findings highlight the need for targeted interventions to address discrimination and promote acceptance in older MSM populations, especially in regions where homosexuality may be less accepted. Programs that promote social support and build resilient coping mechanisms may also be beneficial in reducing loneliness and improving mental health outcomes in this population [43].

48. Chan ASW, Leung LM, Li JSF, Ho JMC, Tam HL, Hsu WL, Iu ANOS, Tang PMK and Yan E (2022) Impacts of psychological wellbeing with HIV/AIDS and cancer among sexual and gender minorities: A systematic review and meta-analysis. Front. Public Health 10:912980. doi: 10.3389/fpubh.2022.912980

49. Alex Siu Wing Chan. (2023) RuPaul’s Drag Race: A Cultural Phenomenon That Challenges Gender Norms and Sparks Conversations Across Borders. Journal of Homosexuality 0:0, pages 1-4.

### Limitation

This study acknowledges potential limitations stemming from its focus on three distinct cultural contexts (Hong Kong, Taiwan, and the PRC) with varying attitudes towards sexual minorities, which might influence participants’ perceptions of discrimination, social acceptance, and their consequent impact on psychological well-being. Furthermore, the study does not comprehensively explore additional contributing factors to the psychological well-being of older MSM, such as their access to healthcare services, the extent of available social support networks or experiences specifically related to aging and associated health concerns. These unexplored variables may play significant roles in shaping the overall mental and emotional well-being of older MSM but were not examined within the scope of this research.

The potential impact of the lifetime recall window utilized in our study. This extended temporal scope for recalling past experiences was considered in terms of its potential influence on participants’ responses. While the lifetime recall window allowed for a comprehensive understanding of participants’ experiences, it may have introduced a degree of recall bias, as memory of events over an entire lifetime could be subject to distortion or selective recall. This extensive temporal scope was chosen to capture participants’ experiences throughout their lives. However, we acknowledge that this approach introduces the possibility of recall bias. Participants may not have engaged in relationships with other males since adolescence or for an extended period, potentially affecting the accuracy and reliability of their recollections. Consequently, the findings of our study should be interpreted in light of this potential source of bias, highlighting the importance of cautious interpretation and consideration of the recall window’s effects on the robustness of our conclusions.

Moreover, this study is rooted in the absence of detailed information regarding the translation process of the 9-item Discrimination and Self-Stigma Evaluation Scale and Perceived Acceptance Scale (PAS). This omission hampers the transparency and comprehensive understanding of the psychometric properties of the instruments employed in the study. Lastly, the recruitment strategies, although successful in reaching our desired demographic amid the pandemic, might impact the generalizability of our results. The utilization of convenience and snowball sampling methods may introduce selection bias, thereby constraining the broader applicability of our findings.

## Conclusion

Our study found that older MSM experience discrimination and lower levels of social acceptance, particularly in the PRC and Taiwan, which can lead to poor psychological well-being. Specifically, we found that the participants reported lower general health, higher levels of discrimination, and lower levels of perceived acceptance, particularly from family members. These findings suggest that discrimination and lack of social acceptance can contribute to social isolation and poor mental health among older MSM. It highlights the need for interventions aimed at reducing stigma and improving social acceptance and support for this vulnerable population. Health and social services should be tailored to meet the specific needs of older MSM, particularly in the areas of mental health and social isolation. Ultimately, creating a more inclusive and supportive environment for older MSM is crucial in promoting their well-being and quality of life.

## Data Availability

All data generated or analysed during this study are included in this published article.
